# Restoration of Full-Length SMN Promoted by Adenoviral Vectors Expressing RNA Antisense Oligonucleotides Embedded in U7 snRNAs

**DOI:** 10.1371/journal.pone.0008204

**Published:** 2009-12-08

**Authors:** Till Geib, Klemens J. Hertel

**Affiliations:** Department of Microbiology and Molecular Genetics, School of Medicine, University of California Irvine, Irvine, California, United States of America; Centre de Regulació Genòmica, Spain

## Abstract

**Background:**

Spinal Muscular Atrophy (SMA) is an autosomal recessive disease that leads to specific loss of motor neurons. It is caused by deletions or mutations of the *survival of motor neuron 1* gene *(SMN1)*. The remaining copy of the gene, *SMN2*, generates only low levels of the SMN protein due to a mutation in *SMN2* exon 7 that leads to exon skipping.

**Methodology/Principal Findings:**

To correct *SMN2* splicing, we use Adenovirus type 5–derived vectors to express *SMN2*-antisense U7 snRNA oligonucleotides targeting the *SMN* intron 7/exon 8 junction. Infection of SMA type I–derived patient fibroblasts with these vectors resulted in increased levels of exon 7 inclusion, upregulating the expression of SMN to similar levels as in non–SMA control cells.

**Conclusions/Significance:**

These results show that Adenovirus type 5–derived vectors delivering U7 antisense oligonucleotides can efficiently restore full-length SMN protein and suggest that the viral vector-mediated oligonucleotide application may be a suitable therapeutic approach to counteract SMA.

## Introduction

Spinal Muscular Atrophy is a neurodegenerative disease that is caused by mutations or deletions in the *survival of motor neuron 1 (SMN1)* gene [1]. Chromosome 5q13 carries two nearly identical copies of the *SMN* gene, *SMN1* and *SMN2*. Both genes code for the same protein, but a C to T transition in the 5′ region of *SMN2* exon 7 results in an altered splicing pattern, where the predominant product lacks exon 7 [2]. Proteins translated from these mRNAs are unstable and not functional. Only 20% of the *SMN2* transcripts contain exon 7 and code for functional full-length SMN protein [1]. It is believed that low amounts of functional protein derived from the *SMN2* gene are sufficient for most tissues in the developing organism, but not for motoneurons where the SMN protein is expressed at about 100-fold higher levels during embryonic development [3], [4]. The specific function of SMN in motoneurons is not yet fully understood, but the disease might be linked to deficiencies in pre-mRNA splicing in developing motoneurons [5], [6], [7], [8], [9]. The 38 kDa SMN protein, which is part of the SMN complex, has been shown to play a major role in the biogenesis and recycling of snRNPs, essential components of the spliceosome [7], [10], [11].

**Figure 1:**
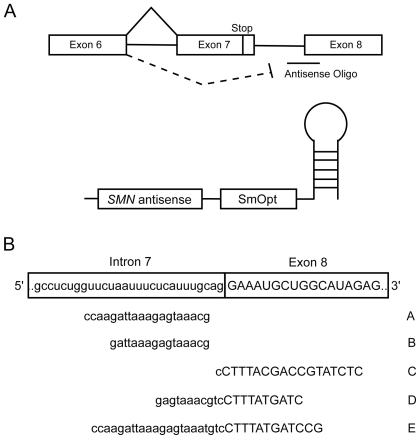
Antisense U7 snRNA strategy and selection of antisense oligonucleotides for Adenovirus delivery. (A) The modified U7 snRNA contains a sequence complementary to the 3′ splice site of *SMN* exon 8. In addition, the wild-type murine U7 Sm binding sequence was replaced with the human consensus Sm binding sequence (SmOpt) to inactivate target cleavage [15]. (B) Nucleotide sequence and target location of the five different antisense oligonucleotides chosen for Adv-5 vector-derived delivery.

To overcome the imbalance in the *SMN2* splicing pattern, we designed antisense RNA oligonucleotides to block the 3′ splice site of *SMN* exon 8. In the presence of these antisense molecules exon 7 inclusion can be induced [12], even when these RNA oligonucleotides were expressed in the context of murine U7 snRNAs [13] (Figure 1A). The native U7 snRNP particle contains an RNA molecule that base pairs with histone pre-mRNA [14]. We exchanged the anti-histone base pairing sequence with an anti-*SMN* exon 8 antisense sequence. Furthermore, the murine Sm binding sequence was replaced with the human Sm binding sequence to avoid cleavage of the targeted sequences [14], [15].

In this study, we use Adenovirus type 5-derived expression vectors to deliver modified U7 snRNAs containing *SMN* exon 8 antisense sequences. We demonstrate that expression of these snRNAs in tissue cultures transiently transfected with *SMN2* minigenes results in increased levels of mRNA transcripts including *SMN* exon 7. Infection of SMA type I patient derived cells with the U7 antisense oligonucleotide expressing vectors leads to elevated exon 7 inclusion ratios as well as increased levels of full-length SMN protein.

## Materials and Methods

### Generation of adenovirus type 5 vectors

U7-antisense RNP encoding sequences were amplified by PCR from previously generated U7-antisense cDNAs [13] and cloned into the pLAD shuttle vector [16]. The resulting shuttle vector constructs were co-transfected with a pJM17 vector containing the AdV-5 viral genome lacking the E1 gene into HEK 293 Adv E1 transgenic cells. This co-transfection allowed homologous recombination of the U7-antisense sequence into the viral genome and production of viral particles. Cells were harvested 10 days post transfection. To purify virus, cells were frozen/thawed 3 times and subsequently sonicated. Virus stock concentrations were determined by plaque assay.

### Determination of exon 7 inclusion levels

For analysis of U7 antisense snRNP effects on *SMN1* and *SMN2* splicing, HeLa cells were passaged in 10% FBS media and transfected with cDNAs encoding either minigene. 24 hours post transfection cells were transformed with adenoviral vectors expressing the different U7 antisense snRNP sequences or with control vectors at an MOI of 20. Cells were passaged for 7 days. The ratio of exon 7 containing transcripts versus those lacking exon 7 was determined by RT-PCR after extraction of total RNA from transfected cell cultures using minigene specific primers. Quantitation of gel purified PCR products was carried out using the BioRad Quantity One software. The effect of U7 antisense snRNPs on endogenous *SMN* transcripts was evaluated in Spinal Muscular Atrophy type 1-derived patient fibroblast cells (Coriell Institute GM 3813) and compared to fibroblast cell cultures derived from the patient's healthy mother (Coriell Institute GM3814). Cells were grown in DMEM media containing 10% FBS for 24 hours before transformation with U7 antisense snRNP expressing Adv-5 vectors or control virus not expressing antisense sequences. After transformation, cell cultures were grown in 2% FBS media. Ten days post infection cells were harvested and total RNA was extracted for semi-quantitative RT-PCR analysis. An exon 6 forward and exon 8 reverse primer set was used for amplification of *SMN* transcripts.

### Analysis of SMN protein expression

Adv-5 transformed cell cultures or un-transformed control cells were harvested 10 days post transfection and analyzed for SMN protein expression levels by quantitative western blotting. For detection of the SMN protein a BD biosciences monoclonal antibody against SMN was used. Expression levels were normalized to β-Actin expression levels using an anti-Actin monoclonal antibody (Sigma). Quantification was performed using Bio-Rad's Quantity one software.

Quantification of SMN protein positive Gemini of Cajal Bodies was performed by immunofluorescence analysis. Patient derived fibroblasts or control cells were grown on cover slips and transformed with U7 snRNP expressing vectors or control vectors 24 hours later. Cells were subsequently grown in DMEM media with 2%FBS for 10 days. For analysis the cell cultures were fixed with 3% paraformaldehyde. SMN localization in nuclear gems was visualized with a monoclonal anti SMN antibody (BD Biosciences) and a Cyanine 3-labeled secondary antibody (sigma). Gem-positive cells were counted in multiple experiments for statistical analysis.

## Results

**Figure 2:**
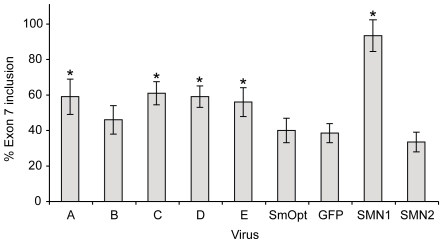
Effect of antisense U7 snRNAs delivered by Adv-5 vectors on *SMN2* minigenes. HeLa cells were transiently transfected with either *SMN2* or *SMN1* minigenes spanning exons 6 through 8. 12 hours post transfection tissue cultures were transduced with Adv-5/antisense U7 snRNAs or control vectors (SmOpt, GFP) at a MOI of 20. The bar summarizes exon 7 inclusion levels observed from 7 independent experiments. Standard deviations are shown for each series. An asterisk above bars indicates a statistically significant difference between the experimental series and the SmOpt control series. A statistically significant difference between series is defined by p-values <0.005.

To make the U7 snRNA methodology more amendable for gene therapy approaches, we generated Adenovirus type 5-derived vectors that constitutively express modified U7 snRNAs. Based on our previous findings, we selected 5 different anti-*SMN* sequences that demonstrated promising effects on upregulating exon 7 inclusion (Figure 1B) [13]. To determine the efficacy of these viral vectors, HeLa cells were transfected with *SMN2* minigenes and subsequently transduced with anti-*SMN* U7 snRNA expressing Adenovirus. In agreement with our previous studies, all vectors expressing an anti-*SMN* U7 sequence resulted in an increase in exon 7 inclusion levels from 33% up to 60% (Figure 2). With the exception of vector B all of these increases are statistically significant (p-values ≤0.005) when compared to uninfected cells or cells that were infected with control vectors either expressing U7 particles that include an antisense oligonucleotide sequence against the unrelated histone mRNA or expressing GFP. Based on multiple experiments, anti-*SMN* U7 snRNA vector C was most efficient in upregulating exon 7 containing transcripts, whereas vector B had the least effect. Transduction with control vectors resulted in a reduced and not highly significant increase in exon 7 inclusion (40% exon 7 inclusion, p-value = 0.05 for SmOpt and 38% exon 7 inclusion, p-value = 0.11 for GFP). Taken together, these results indicate that delivery of antisense U7 snRNAs by Adenovirus type 5-derived vectors has a similar efficacy in modulating the splicing pattern of *SMN2* minigenes as transient transfection of antisense U7 snRNA expressing cDNAs.

**Figure 3:**
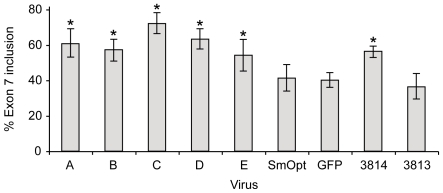
Evaluation of endogenous *SMN* splicing patterns in SMA type I patient-derived fibroblasts. SMA type I 3813 cells were transduced with Adv-5 vectors expressing antisense U7 snRNAs or control vectors (GFP, SmOpt) at a MOI of 20. Cells were passaged for 10 days in 2% FBS medium, and then harvested for RNA extraction. The bar graph gel image summarizes the levels observed from 8 independent experiments. Standard deviations are shown for each series. An asterisk above bars indicates a statistically significant difference between the experimental series and the SmOpt control series. A statistically significant difference between series is defined by p-values <0.005.

To investigate whether antisense U7 snRNAs can also modify the splicing pattern of the endogenous *SMN2* gene, we used a SMA patient fibroblast cell line (GM3813). These cells have no functional *SMN1* gene and only two copies of the *SMN2* gene [17]. Therefore, all SMN protein detected in these cells, full-length and truncated, is generated from the *SMN2* gene copies. In contrast, higher levels of functional SMN protein are produced in GM3814 cells, a fibroblast cell line derived from the mother of the SMA patient, due to the presence of one intact copy of the *SMN1* gene. The expression difference between these cell lines has been used as a benchmark to define sufficient *SMN* expression because the carrier does not show defects in motoneuron outgrowth. The patient-derived cell line GM3813 was infected with Adv-5 vectors encoding different antisense U7 oligonucleotides at a MOI of 20 and GM3814 cells were used as a reference (Figure 3). All cell cultures infected with antisense U7 snRNA-expressing vectors displayed significantly increased ratios of exon 7 containing splice products compared to the uninfected patient cell line or the control GFP and SmOpt vectors (p-values ≤0.005), with sequences C and D being the most efficient (Figure 3). In contrast, cell cultures transduced with Adv-5 control vectors GFP and SmOpt showed no statistically significant increase in exon 7 inclusion levels compared to uninfected GM3813 cells (Figure 3) (p-values ≥0.4). We conclude that anti-*SMN* U7 snRNA expression in SMA type I fibroblasts can increase the ratio of exon 7 inclusion to levels comparable to healthy carrier individuals.

**Figure 4:**
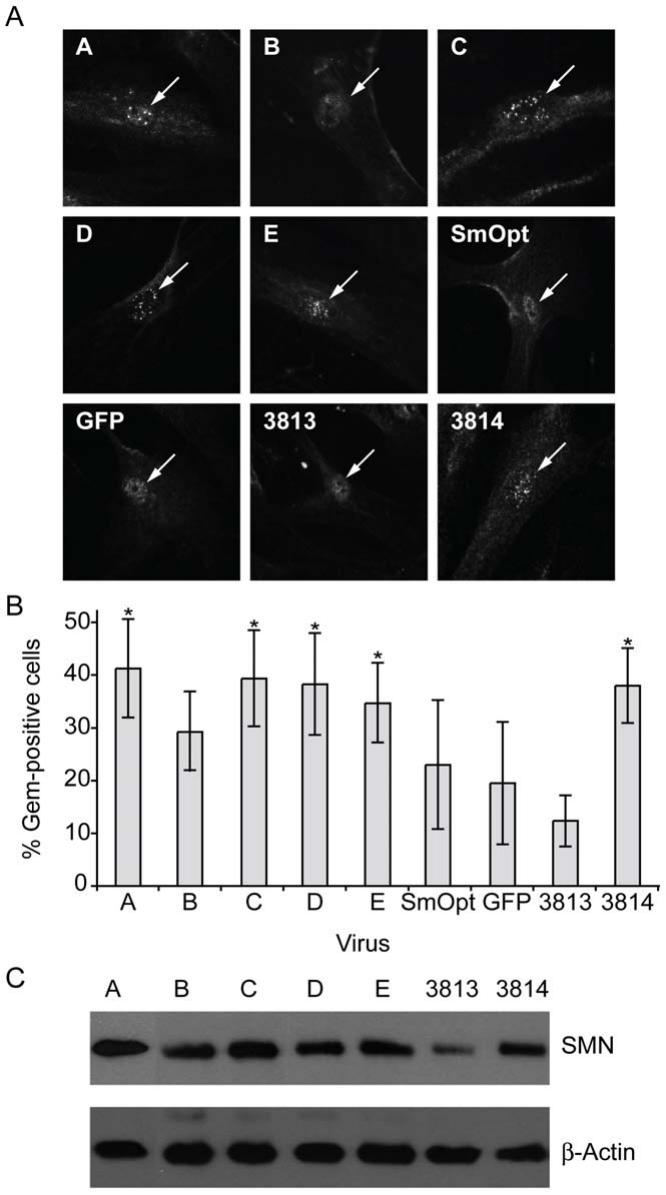
Determination of changes in functional protein levels following Adv-5/antisense U7 snRNA infection. (A) SMA type-I fibroblast cells (G3813) were transduced with Adv-5 vectors either expressing antisense U7 snRNAs or control vectors expressing SmOpt or GFP. The cell cultures were passaged for 10 days in 2% FBS medium, fixed with formaldehyde, and analyzed using immunofluorescence. As an indicator for SMN levels the fraction of nuclear gem-positive cells was determined by fluorescence microscopy. Cell nuclei are marked with white arrows. The identity of the Adv-5 vectors is indicated on the left upper corner of each panel. (B) The bar graph summarizes gem counts from 5 independent and blinded experiments. Standard deviations are shown for each series. An asterisk above bars indicates a statistically significant difference between the experimental series and the SmOpt control series. A statistically significant difference between series is defined by p-values <0.05. (C) Western blot analysis of cell cultures infected with AdV5/U7 antisense snRNPs. G3813 cells were transduced with AdV5/antisense U7 snRNPs at an MOI of 20. The expression levels were normalized to β-Actin using an anti β-Actin monoclonal antibody. Relative SMN protein levels were determined by SMN staining using a monoclonal anti SMN antibody.

Previous studies demonstrated that the protein produced from the truncated transcript of the *SMN2* gene is unstable and not fully functional [18], [19], [20], as indicated by the presence of SMN in nuclear gems, a concentrated compartment of the SMN complex [21]. Compared to healthy individuals, most SMA type I patient-derived fibroblast cells have no or very limited amounts of SMN containing nuclear gems [22]. To address the question whether infection by Adv-5 antisense U7 snRNAs results in increased levels of full-length SMN protein, we determined the percentage of gem-positive cells in Adv-5/U7 snRNA transduced tissue cultures (Figure 4A). As a reference, the percentage of gem-positive cells was also determined in uninfected GM3813 patient and GM3814 mother cell cultures. Ten days after infection, all anti-*SMN* U7 expressing cell cultures except those infected with vector B displayed statistically significant increases in nuclear gem counts compared to patient cell lines that were infected with SmOpt or GFP control vectors (Figure 4B) (p-values ≤0.05 when compared to SmOpt). As expected from their efficiency to upregulate exon 7 inclusion, anti-*SMN* U7 sequences A, C and D showed the highest percentage of gem-positive cells at levels comparable to those observed in uninfected parental control cells (3814). These data indicate that full-length SMN protein expression was elevated to the level of a healthy individual. For further validation we performed western blot analysis to measure SMN protein levels in cell cultures treated with antisense U7 snRNPs. Relative levels of SMN protein were determined by normalization to β-Actin levels. Our results show a noticeable increase of total SMN protein levels in cells treated with antisense U7 snRNPs over uninfected cell cultures (Figure 4C). We conclude that antisense U7 snRNAs delivered by Adv-5 vectors induces the expression of functional SMN protein in cell cultures.

## Discussion

Using the Adenoviral vector tool enabled us to study the effects of anti-*SMN* U7 snRNAs on endogenous *SMN* expression in SMA type I patient fibroblasts. These cells have no functional *SMN1* gene and contain only two copies of *SMN2* [17]. Transduction of these cells with antisense U7 snRNA-expressing vectors resulted in elevated ratios of transcripts containing exon 7 (Figure 3). In earlier studies we showed that skipping of exon 7 results from a competition between the 3′ splice sites of intron 7 and 8. Significantly, targeting the intron 8 3′ splice site with antisense oligonucleotides resulted in preferred use of the intron 7 3′ splice site and, thus, in elevated levels of exon 7 inclusion [12]. The fact that full-length SMN protein levels were restored to those in a healthy carrier (Figure 4) supports the notion that the antisense approach can be considered as a potential therapeutic strategy for the treatment of SMA. Application of either a nonspecific antisense oligonucleotide coding U7 particle or a particle encoding GFP resulted in a small increase of exon 7 inclusion rates (Figure 2 and Figure 4). This is most likely due to a nonspecific effect of the AdV-5 vector, because application of theses modified U7 particles by transfection does not result in such an increase [12], [13].

The presence of nuclear gems is a commonly accepted measure to quantify levels of SMN protein in a cell, as SMN is an essential component of these nuclear structures [21], [23]. Immunofluorescence staining of SMN revealed that the percentage of gem-positive cells was significantly increased after transduction with Adv-5/antisense U7 snRNAs (Figure 4). These observations and western blotting demonstrated that U7 antisense oligonucleotides blocking the exon 8 3′ splice site of *SMN2* can restore SMN levels in patient cell lines to those observed in carrier cell lines. However, while the increase in gem counts demonstrates proper nuclear localization, it is still unclear whether upregulating SMN levels restore normal functionality.

Embedding the antisense sequence in an U7 snRNP complex has several advantages over application of naked RNAs. First, RNA splicing takes place in the nucleus where U7 snRNP particles are located. Second, binding of U7-specific proteins and 5′ capping of the U7 snRNA also ensures an increased half-life. Third, the presence of U7 snRNA transgenes guarantees continuous expression. These considerations make the use of antisense oligonucleotides-embedded U7 snRNAs an attractive approach, as was highlighted recently by the successful application to modulate pre-mRNA processing in the dystrophin [24], [25], SMN [26], and the thalassemic β-globin genes [27]. Additional advantages can be gained by delivering these antisense U7 snRNAs within viral vectors. We were able to study the effects of antisense sequences in patient-derived fibroblast cell lines, whereas transfection of these cells usually results in toxicity, even when cationic transfection reagents are used. Infection with viral vectors reaches the majority of cells analyzed, while the efficiency of transient transfections is limited. Importantly, adenoviruses can infect a broad range of different mammalian cell lines and animal species without integrating into the host cell genome [28]. Adv has a tropism for neuronal cells and can be used to deliver transgenes to the central nervous system [29], [30], [31]. Furthermore, Adv-5 is replication deficient. The viral E1 gene is deleted from the vector genome, thus the virus cannot replicate and can only be grown in human embryonic kidney cells that are transformed with the viral E1 gene. Accordingly, Adv-5 vectors have been proposed for multiple gene therapy strategies and some have been approved for clinical trials by the FDA [28], [31].

It is not fully understood how the loss of the housekeeping gene *SMN1* leads to the selective loss of motoneurons in the spinal cord. Recent studies have shown that in mouse spinal cords the rate of ribonucleoprotein assembly is the highest during myelination of the central nervous system. SMN plays an essential role in this assembly process [7]. In a zebrafish model of SMA it was shown that the addition of purified snRNPs could rescue motoneuron impairment induced by the loss of SMN [5], [32]. Accordingly, reduced snRNP levels in SMA mice or SMA fibroblast cell lines have been proposed to result in a general pre-mRNA splicing defect [8], [33]. Together these data indicate that the window for effective treatment or prevention of SMA might be very early during embryonic development. Future efforts will concentrate on evaluating the anti-*SMN* U7 snRNA Adv-5 gene therapy approach in an animal model of SMA. Importantly, the administration of the virus during various stages of fetal development could pinpoint the optimal time point for initiating treatment for SMA.
